# Epileptic Seizure Cycles: Six Common Clinical Misconceptions

**DOI:** 10.3389/fneur.2021.720328

**Published:** 2021-08-04

**Authors:** Philippa J. Karoly, Dean R. Freestone, Dominique Eden, Rachel E. Stirling, Lyra Li, Pedro F. Vianna, Matias I. Maturana, Wendyl J. D'Souza, Mark J. Cook, Mark P. Richardson, Benjamin H. Brinkmann, Ewan S. Nurse

**Affiliations:** ^1^Seer Medical, Melbourne, VIC, Australia; ^2^Department of Biomedical Engineering, The University of Melbourne, Melbourne, VIC, Australia; ^3^School of Neuroscience, Institute of Psychiatry, Psychology & Neuroscience, King's College London, London, United Kingdom; ^4^Faculty of Medicine, University of Lisbon, Lisbon, Portugal; ^5^Department of Medicine, St Vincent's Hospital Melbourne, The University of Melbourne, Melbourne, VIC, Australia; ^6^Bioelectronics Neurophysiology and Engineering Lab, Department of Neurology, Mayo Clinic, Rochester, MN, United States

**Keywords:** seizure cycles, epilepsy, multiday cycles, EEG, wearables

## Introduction

The propensity for seizures to follow circadian and multiday (i.e., weekly, monthly, or seasonal) rhythms has been documented for centuries (1, 2). More recent findings from chronically recorded EEG in both human and animal studies have further elucidated the existence of multiday rhythms governing seizure timing and rates of epileptic activity [see (3) for a recent review]. These multiday epileptic cycles are found to be prevalent in a number of studies, including from implantable devices (4–7), electronic seizure diaries (8, 9), and wearable monitoring (10). It is becoming clear that multiday seizure cycles are important phenomena in epilepsy, with many implications for seizure management and more broadly in interpreting research studies and clinical trials (11–13).

The integration of knowledge of seizure cycles into clinical practise is in an early phase, with some theoretical studies demonstrating the utility of seizure risk forecasts (14–16) or scheduling diagnostic testing (9) based on multiday cycles. However, several barriers remain before seizure cycles can be widely adopted in clinical management. One barrier is the knowledge gap between data scientists and clinicians. Cycles are measured and described using circular statistics and frequency analysis, making the explanation of the presence and strength of cycles technically complex. It is yet to be determined at what strength a cycle can be deemed to be clinically relevant for making treatment or monitoring decisions. Much progress has been made converging the clinical and engineering aspects of epilepsy, however seizure cycles present new concepts in seizures and epilepsy that need to be clearly interpretable by clinicians (13, 17). This review aims to bridge several gaps that commonly arise between data science and clinical practise.

## Misconception 1: Seizure Diaries Are Too Noisy to Infer Cycles

It is well-known that self-reported events (i.e., seizure diaries) are unreliable (18), nevertheless diaries remain the primary method of monitoring disease burden in epilepsy. Notably, it was through the meticulous recording of patients' seizures that early neurologists first recognised multiday seizure cycles (1, 2, 19, 20). These centuries-old hand charting of seizure occurrence resulted in critical insights into multiday periodicity in epilepsy that still await explanation. For instance, in J.R. Reynolds' seminal textbook from 1861 he observed:

“*it appears, therefore, that although regular periodicity is rarely observed in epilepsy, and is entirely absent in some cases, yet than in the majority of cases there is an approximation to periodicity, and that the recurrence of attacks occupies a somewhat marked relation to the natural divisions of time; such as the day the month, and fractional parts of the month”*

In an earlier 1746 treatise, Richard Mead also noted that, “great regard must be had to the times in which the paroxysms most usually return, in order to effect a cure.” Evidently, diary records have been of immense value in establishing the existence of multiday seizure cycles.

On the other hand, advances in chronic EEG recording have shown that seizure cycles are more reliably estimated by continuously monitoring biomarkers of excitability (5). For instance average rates of interictal epileptic activity (4), the variance and autocorrelation of EEG (15), and even average heart rate (21) all show multiday cycles that are more robustly predictive of seizure likelihood than looking at past seizure times. Additionally, a recent comparison of seizure diaries and epileptic activity captured from chronic sub-scalp recording systems (22) showed discrepancies between the cyclic distributions of self-reported and electrographic events, even when diaries were relatively accurate (23). This is perhaps not unexpected given many seizures are not recognised by patients, particularly those occurring in sleep.

Nevertheless, for some people, self-reported events can be used to track seizure cycles (see case study). One recent study found the distributions of seizure diary events and electrographic seizures were not significantly different for nine out of 15 subjects, suggesting that although participants vastly underreported the total number of seizures, the underlying multiday cycles can still be determined for some individuals (8). In a retrospective analysis of the same cohort, forecasts based on self-reported seizures were accurate for electrographic events for four out of eight participants (8). It should be noted that this small cohort may not be representative of the broader diary-using population, and may in fact be more accurate in order to have been selected for this study (24). Another retrospective forecasting study found multiday cycles in the rates of interictal epileptic activity corresponded to self-reported seizure risk for most individuals (14). Similarly, a validation study found cycles measured from seizure diaries corresponded to the occurrence of epileptic activity during ambulatory EEG monitoring (9). Machine learning approaches have also been successfully deployed to forecast seizure risk from electronic diaries (25, 26).

On the whole, although advances in tracking continuous cyclic biomarkers can provide more reliable measures of seizure risk (27), seizure diaries should not be discounted as a useful tool to monitor individuals' seizure cycles and seizure risk within clinical settings. Patient diaries are likely to remain a cornerstone of clinical monitoring, and should be incorporated to characterise seizure cycles where possible, in particular for low risk applications such as scheduling EEG monitoring (9). Further work is needed to characterise the relationship between cycles of interictal epileptic activity and self-reported events, as well as to identify the subset of individuals whose self-reported seizure cycles align with their electrographic events.

### Case Study: Self-Reported and Electrographic Seizures Correspond to Underlying Cycle of Epileptic Activity

**Figure 1:**
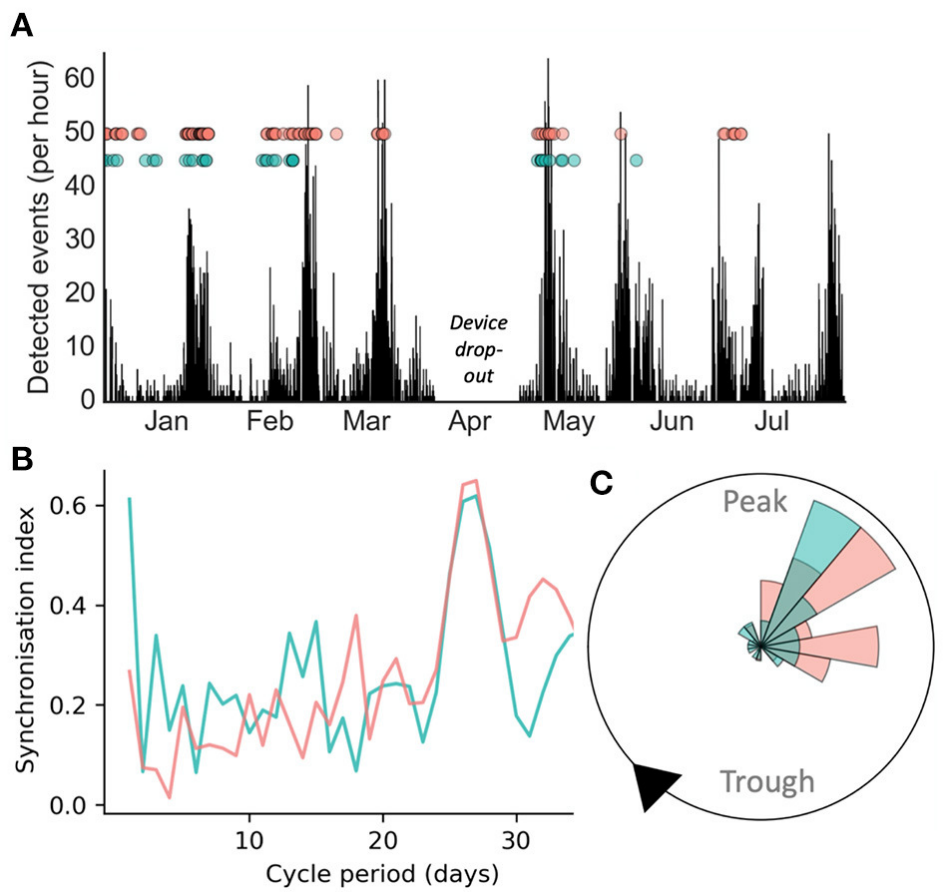
Case study of electrographic seizure detection from sub-scalp EEG (red) and seizure diary self-reported events (green) over >6 months in an individual patient. **(A)**: counts of detected epileptiform events over time, with seizures shown as dots. **(B)** Synchronisation index measured from event times at different fixed cycle periods showed a similar peak around 28-days for both EEG and self-reported seizures **(C)** A polar plot demonstrating that both diary and EEG seizures show similar phase distributions with respect to the underlying 28-day cycle.

Figure 1 presents a case study of an individual adult female diagnosed with seizures secondary to a periventricular nodular heterotopia, refractory to polytherapy, implanted with a chronic sub-scalp EEG recording system (Epi-Minder “Minder” system) (28). Event labels and cycles of epileptic activity were detected using the method described in Stirling et al. (28). It can be seen that self-reported and electrographic seizures appeared to align to the same underlying cycle of interictal epileptic activity (Figure 1A). Cycles were also measured from seizure times alone (rather than the continuous rate of interictal epileptic activity), using the method described in Karoly et al. (8). Using this approach, both self-reported and EEG seizures showed strong alignment to a fixed underlying cycle of 28-days (Figure 1B), which agreed with the cycle of interictal epileptic activity. The phase distributions of self-reported and electrographic events with respect to the 28-day cycle of interictal epileptic activity (Figure 1C) were not significantly different (*p* > 0.05 using a Kolmogorov-Smirnov test for equality). Therefore, this individual was able to self-report her seizures reliably enough to estimate the high-risk periods of her underlying multiday cycle, in agreement with validated electrographic seizures.

## Misconception 2: Weekly Cycles Are Driven by Weekday Behaviours

Multiday seizure cycles are consistently found at about-weekly (5–9 day) and about-monthly (28–32 day) periods (4, 7, 29), which is often attributed to behavioural (weekday) or catamenial effects. For instance, a large cohort study found multiday cycles in 60% of people with epilepsy, with common periods 7, 15, 20, and 30 days (29). Cycles of around 30 days were as common in men as in women, suggesting catamenial influences are probably not explanatory (see subsequent section). Research suggests that behaviours such as diet, alcohol consumption, sleep and wake times and exposure to stress impact seizure risk in an individualised manner (30, 31) and this evidence has led to hypotheses that behavioural triggers underpin multiday seizure cycles. Regular social routines are commonly proposed as the source of weekly seizure cycles however there is no clear evidence linking cyclic behavioural patterns to cycles of seizure susceptibility. Conversely, multiday weekly, and monthly epileptic rhythms are only occasionally modulated by fixed cycles, such as weekdays or day of month (3, 32).

**Figure 2:**
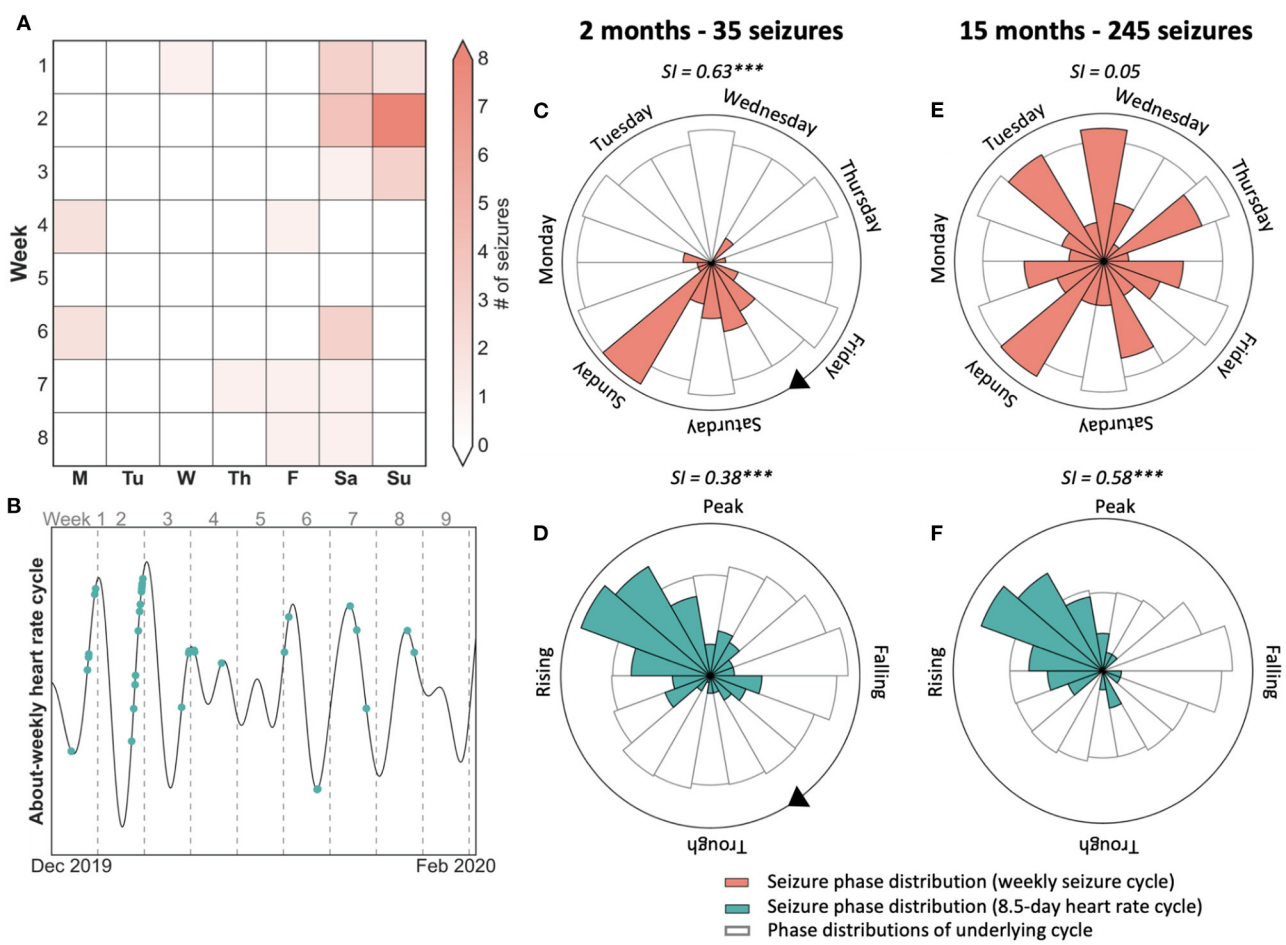
Case study of 7-day weekly seizure cycle and 8.5-day heart rate cycle showing that seizures were not modulated by weekly behaviour. **(A)** 8-week seizure diary showing days that seizures occurred, suggesting a weekly seizure cycle. **(B)** 8.5-day heart rate cycle of the same period as (a), with seizures shown in green. Seizures tend to occur on the rising phase of the cycle, with a few exceptions. **(C,D)** Polar plots demonstrating the location of seizures with respect to the 7-day seizure (red) and 8.5-day heart rate cycle (green) after two months and 35 seizures. Seizures were more strongly locked to the 7-day week. **(E,F)** Polar plots demonstrating seizure timing on the seizure and heart rate cycle after 15 months and 245 seizures. Seizures were no longer locked to the weekly cycle but remained strongly locked to the 8.5-day heart rate cycle. SI, Synchronisation index value with significance according to Omnibus test (***p <= 0.001).

Nevertheless, several studies have noted an effect of weekdays on seizure occurrence, suggesting that the weekly routine leads to some entrainment of naturally occurring rhythms. A large study of seizure diaries found that 7-day seizure cycles were significant in about 20% of people with epilepsy (7). Although, at the population level, there was no clear preference for seizures to occur on a particular day or part of the week. Rao et al. found a weak, significant preference for fewer electrographic seizures on Sunday, similar to the results of Ferastraoaru et al. of seizure diaries (32, 33). On the other hand, alignment of weekly seizure cycles to the 7-day week is often transient (32) (see also case study, Figure 2), suggesting there are more prominent endogenous physiological mechanisms that modulate seizure occurrence with approximate weekly rhythms.

It has been hypothesised that endogenous weekly cycles do influence mammalian physiology, with evidence of such cycles affecting the cardio-respiratory, immune, and endocrine systems (34). A recent study found people with epilepsy showed weekly fluctuations in resting heart rate that were related to their seizure occurrence (21). Similar to epileptic activity, heart rate cycles were found at patient-specific periods of between 5 and 9 days and not necessarily linked to weekdays. Overall, the body of evidence suggests that, for some individuals, the 7-day week acts as a synchronizer, rather than a driver, of weekly seizure cycles.

### Case Study: Weekly Seizure Cycle Not Aligned to the Day of Week

Figure 2 presents a case study of an individual with focal epilepsy who shows their 8-week seizure diary (Figure 2A) to their clinician. After observing the preference for seizures to occur on weekends, the clinician may suspect that this patient's seizures are driven by an after-stress effect, i.e., they occur on the weekend at the end of the stressful working week. The polar plots (Figures 2C,D) validate this belief by showing that seizures have a stronger link to the weekly cycle than to an intrinsic 8.5-day heart rate cycle (Figure 2B). However, after 15 months have passed (Figures 2E,F), seizures are no longer linked to the weekly cycle but remain well-aligned by the 8.5-day heart rate cycle, which is likely to be endogenous in origin. Therefore, based on 2 months of observations (i.e., 7 × 8.5-day cycles) seizures seemed linked to a behavioural driver, although the endogenous long-term pattern of heart rate was more stable over a 15-month observation period.

## Misconception 3: Monthly Cycles Are Catamenial Epilepsy

Despite centuries of data showing that males and females experience about-monthly seizures with similar prevalence (4, 7, 35, 36), there is a persistent view that approximate monthly (i.e., 3–5 weeks) cycles of seizure occurrence in women must be linked to the menstrual cycle. The prevalence of catamenial epilepsy is variously reported to be between 10 and 70% (37). Clinically, catamenial epilepsy is defined as an increase (i.e., 2-fold increase) in seizures during some phase of the menstrual cycle, generally documented by self-report (38). Most studies reporting on the prevalence of catamenial epilepsy are based on short-term analysis of menstrual or hormonal data over just two to three cycles (39). Limited duration studies of several months may not be sufficient to determine whether changes in seizure frequency are significant. Furthermore, monthly seizure cycles can transiently align with the calendar month, despite being more closely linked to underlying cyclic fluctuation in epileptic activity which may not be of exactly one calendar month in duration (32). Therefore, it is possible that monthly cycles of seizure occurrence appear to be linked to menstruation over several months, however the statistical relationship would be abolished after longer term monitoring (see case study).

In contrast, some women with about-monthly seizure cycles may have seizures significantly linked to their menstrual cycle over long-term monitoring. The underlying cause/s of monthly seizure cycles are unknown, and are likely to differ between individuals, with oestrogen or sex hormones playing a role for some individuals. In support of this hypothesis, some studies in animal models have found evidence for the influence of oestrogen on seizure occurrence (40, 41). Furthermore, although a landmark study of hormone treatment for women with suspected catamenial epilepsy did not show a significant effect (42), *post-hoc* analysis revealed a smaller subset of women for whom hormone treatment may have been effective (43). The findings suggest that catamenial effects are just one facet of monthly seizure cycles, rather than being the primary mechanism.

For clinicians, the recognition that about-monthly cycles of seizure activity in women may not relate to underlying menstrual patterns is important in avoiding perhaps futile manipulation of hormonal cycles through medication, as are often proposed by clinicians and patients.

### Case Study: Monthly Seizure Cycle Not Aligned to Menstruation Times

**Figure 3:**
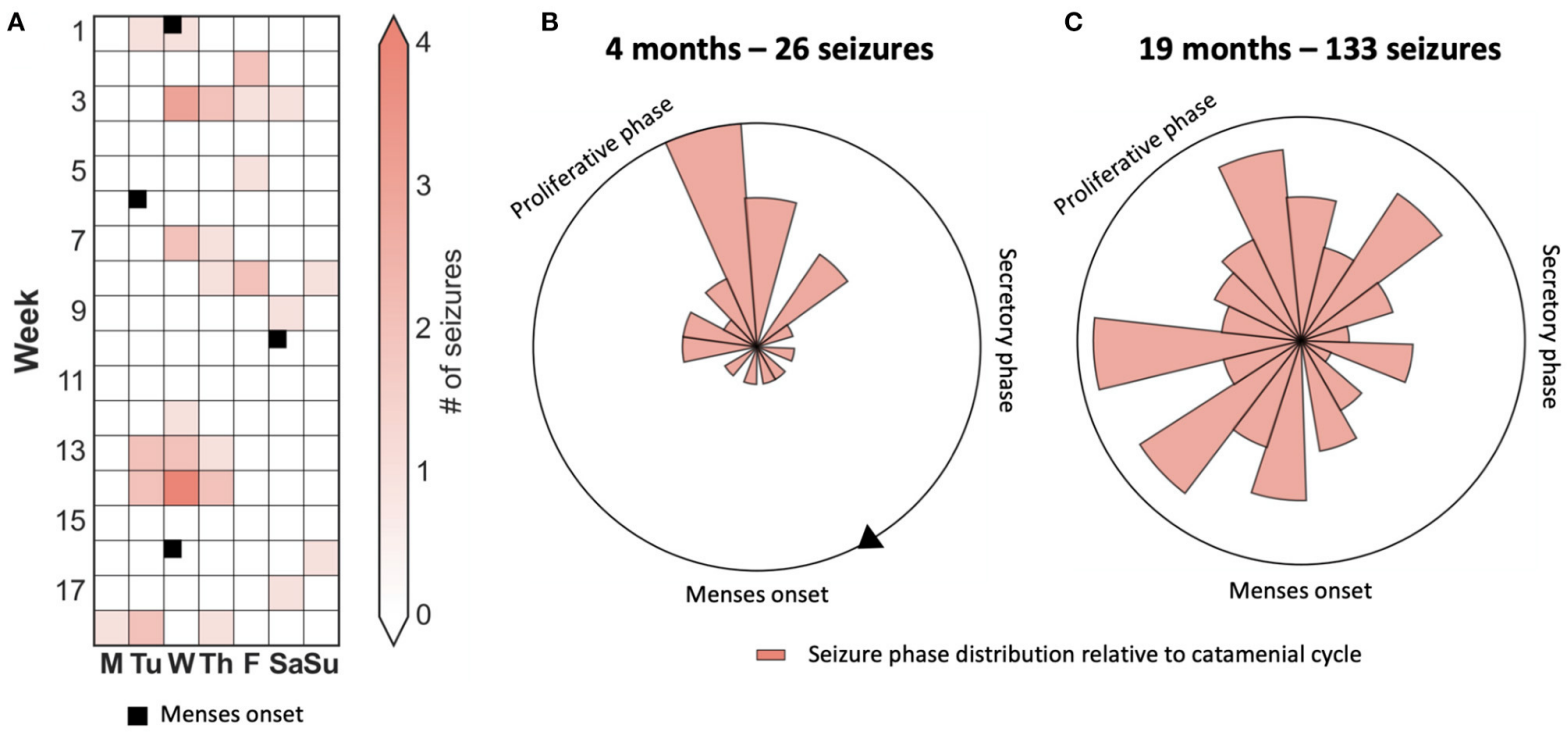
Case study of a catamenial seizure cycle showing that seizures are only transiently aligned to the menstrual phase. **(A)** 4-month seizure diary showing days that seizures occurred, and times of menstruation, suggesting a catamenial seizure cycle. **(B,C)** Polar plots demonstrating the location of seizures with respect to the menstrual cycle after four months and 26 seizures **(B)** and after 19 months and 133 seizures **(C)**.

Figure 3 presents the case of a patient who appeared to have strong phase-locking between seizures and menses when observed over 4 months. However, there was no alignment over an observation period of 19 months. Hence, quantification of cycles using robust statistical techniques over long periods is required to avoid erroneous conclusions.

## Misconception 4: Cycles Are Driven by Medication

The influence of anti-seizure medications (ASMs) has been suggested as a driver of circadian cycles of epileptic activity and seizures, since the precise time at which medication is taken can shift the peak seizure occurrence within a 24-h period (35, 36). Consequently, medications are sometimes touted as a possible driver for longer, multiday rhythms. Although the circadian rhythm of seizure occurrence may be influenced by ASMs, the short half-life of most ASMs reduces the likelihood that medication is driving longer rhythms (44). It is possible that the interactions between multiple ASMs taken throughout the day can result in periods of high and low therapeutic effect and therefore affect seizure likelihood throughout the day. However, there is no clear explanation for how the timing of daily (or more frequent) medication use could drive fluctuations in seizure likelihood that repeat over weeks to months or even seasonally.

Several animal studies have also established the existence of multiday cycles of epileptic activity in the absence of ASM use. A small study in six dogs with naturally occurring canine epilepsy included one dog where a weekly seizure cycle was maintained in the absence of ASM usage (45). Another study in a rodent model of epilepsy found multiday weekly cycles in the epileptic activity of rats who were not treated with ASMs (46). Similarly tetanus-toxin models of temporal lobe epilepsy also show cycles where seizures strongly cluster (47).

It is possible that systematic bias affects when people miss their medications (i.e., on weekends, or when each pack runs out), providing a periodic seizure trigger. Missed medication is one of the most common seizure triggers (31, 48), and clinically significant non-adherence has been reported to affect between 29 and 79% of people with epilepsy (with the variance due to different definitions of what constitutes significant non-adherence) (49). For instance, one study found 66% of people reported missing their medications at least once per month (50). However, while the aforementioned studies identify demographic factors that contribute to medication adherence, to our knowledge no studies have investigated the timing of missed medication, such as whether people are more likely to miss medication on certain days or with any regularity. Sustained periods of medication non-adherence could also be particularly relevant, as cyclic fluctuations in seizure risk may build up over slow timescales of days to weeks (3).

There are no studies that systematically analyse the relationship between ASM usage and cycles in epilepsy. Consequently, it is not possible to discount ASMs as a confounding factor in the detection of seizure cycles. However, some evidence suggests that seizure cycles exist despite medications in humans. The ancient Greeks described approximately monthly cycles in seizures that were thought to be associated with lunar cycles (51), a theory that is purported even today (52). A 1,929 investigation into 66 patients with epilepsy reported three types of circadian distributions over 2,524 seizures (53). The authors concluded that while sedatives (bromide or luminal) reduced the rates of seizures, their effects were negligible in driving the cycles. Soon after, a report into seizures in 110 boys at an epileptic colony over a 10 year period discovered circadian, and weekly, monthly, and even yearly cycles in seizures (36). Similarly, the authors concluded that drugs reduced the number of seizures, but the grouping of seizures were resistant to drugs.

Hence, although systematic studies remain to be undertaken, there is a significant body of historical and pre-clinical models suggesting that ASMs do not drive cycles of seizure activity.

## Misconception 5: cycles Are Only Relevant for Focal Epilepsies

The prevalence of multiday cycles across different epilepsy types, or whether distinct seizure types adhere to different cycles, are commonly asked questions regarding epileptic cycles. Unfortunately, these questions are difficult to answer since contemporary studies have principally identified cycles of epileptic activity from chronic implanted EEG in patients with focal epilepsies. To our knowledge, there are no studies that report on chronic EEG in individuals with genetic generalised epilepsies (GGE), and long-term electrographic correlates are yet to be reported. Historic records of seizure timing may include individuals with GGE, such as the Griffiths and Fox (36) et al. record of two sisters with early onset epilepsy and “curiously alike” seizure cycles around 2 years in duration (36). However, such historic diagnoses are highly speculative and cannot be relied upon. Contemporary diary studies have shown multiday seizure cycles can be measured from self-reported event times in people with GGEs (7, 9), and have corresponding cycles of heart rate activity (21).

Particular generalised epilepsy syndromes are well-characterised as having ultradian cycles of interictal and clinical epileptiform activity in both adults and children (54–58), and similar circadian cycles particularly related to sleep-wake transitions (55, 59, 60). The thalamocortical networks associated with slow-wave sleep are also implicated in the generation of generalised spike-wave discharges (56, 61). Hence, despite a lack of longitudinal EEG to validate patient-specific infradian cycles, there are well-studied circadian and ultradian rhythms present in GGE.

From a network perspective, a focal seizure originates from a single region of the brain (and may or may not generalise), while a generalised seizure is a more inherent property of the overall network where no single foci is identifiable (62). Although overlapping symptomatically, this fundamental difference in generalised seizure generation may mean that electrographic multiday cycles are not present in generalised epilepsies due to the difference in the seizure generation process. We anticipate that the next generation of chronic EEG devices (23, 28, 63) will be able to elucidate any such cycles as they have for focal epilepsies.

### Case Study: Ultradian Seizure Cycles in an Individual With JAE

**Figure 4:**
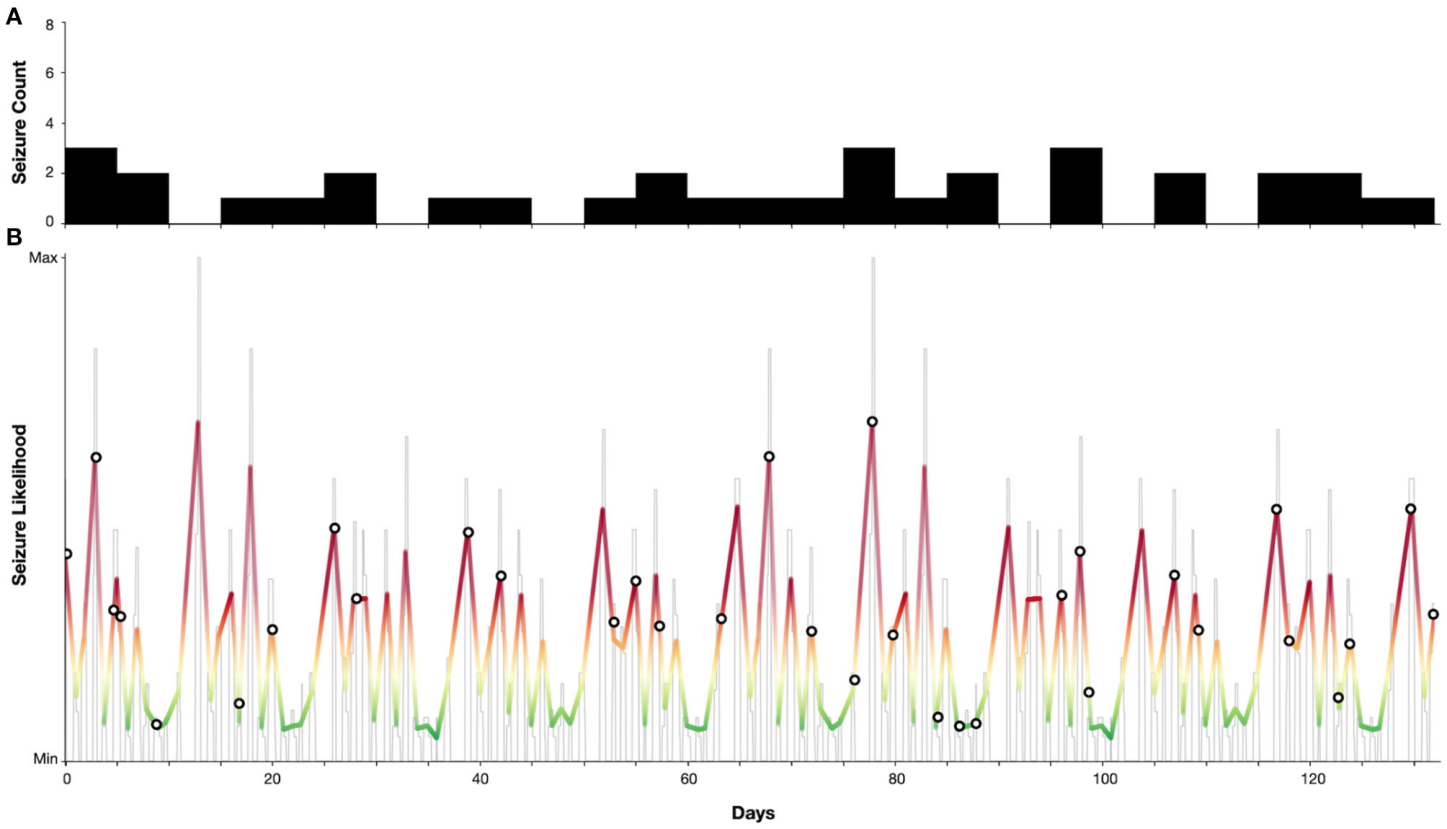
Case study of an individual with juvenile absence epilepsy with 5 and 13-day seizure cycles. **(A)** Self-reported seizure counts (bin-width of 5 days). **(B)** Estimated seizure likelihood (grey—raw likelihood estimate, colour—smoothed likelihood). Markers indicate self-reported seizure occurrences. Note that the majority (79%) of seizures occurred when the seizure likelihood was high.

Figure 4 demonstrates a case study of multiday cycles derived from an electronic seizure diary (Seer app) from an adult woman with a confirmed diagnosis of juvenile absence epilepsy (JAE), with refractory convulsive and absence seizures. Two distinct cycles were identified at 5 and 13 days from a seizure diary with a total duration of 18 weeks. From these cycles a forecast was generated as per (9), resulting in 24% of time spent in high seizure risk and 79% of seizures occurring in high risk. Hence, although generated from self-reported events, this case presents seizure cycles from an individual with a generalised epilepsy capable of producing an accurate forecast.

## Misconception 6: Cycles Are Driven by Epilepsy-Specific Phenomena

To understand the cause/s of multiday seizure cycles, it is necessary to look beyond epilepsy and even beyond neurology. Slow physiological rhythms have been documented across a range of human diseases (64). Episodic psychiatric conditions are suggestive of multiday modulation, including bipolar disorder (65, 66), depression (67, 68), and other psychopathologies (69). In cardiology, blood pressure and heart rate have been found to show endogenous weekly cycles (70), and 7-day and seasonal patterns have been documented for cardiovascular diseases (71–73). As well, immunologists have long recognised weekly cycles governing inflammatory markers in the blood, including antibody production, circulating lymphocytes, and cellular immunity in animal studies (74), which appear to impact individuals' responses to cancer treatment (75, 76).

Although very limited, some studies have also identified physiological cycles with weekly, monthly, and seasonal patterns for healthy individuals. Seven-day rhythms have been studied, to a small extent, in endocrinology. Circaseptan variations in cortisol were found on 20 healthy subjects after sampling thrice weekly for three months (64). Melatonin has also been observed to vary on a weekly basis (77); however, the study was limited to weeklong recordings on a small cohort. Evidence for monthly rhythms in biological phenomena is limited but includes sleep quality (78) and hormone levels, including testosterone (79). Monthly rhythms can also be found in heart rate, but this is likely to be rare; one study mentioned about 3% of people (80). Within populations, weak seasonal cycles have been observed in some physiological states and biomarkers, including human cognition (81), skin temperature (82), and salivary cortisol (83). However, these seasonal changes were most likely related to environmental drivers, such as ambient temperature and photoperiod.

The collective evidence of multiday cycles in other spheres of human physiology and disease hints that similarly long timescale rhythms in epilepsy do not arise only because of epileptogenesis or ictogenesis. Instead, systemic physiological rhythms may combine into complex, individual-specific oscillations that lead to the periodic emergence of pro-ictal conditions. This hypothetical emergence of seizure cycles from an underlying network of oscillators has been outlined in a recent review of circadian molecular oscillations and rhythmicity of epilepsy (84). Ultimately, to understand cycles in epilepsy, it will be critical to understand how many other physiological cycles interact with rhythms of seizures and epileptic activity.

## Conclusion

Cycles of epileptic activity have been documented for centuries, however the recent emergence of technologies such as electronic seizure diaries, wearable physiology tracking devices, and chronic EEG recordings have allowed for their thorough investigation. It is increasingly clear that multiday seizure cycles are highly patient specific. Although some seizure types and epilepsy syndromes are known to have population-wide characteristics with respect to circadian seizure timings, analogous features are only just beginning to be characterised at multiday time scales (7, 29). Further work is required to understand whether demographic or clinical factors may be predictive of cycle periods. Most of the work presented here involved retrospective, exploratory data analysis, hence prospective studies with explicit predefined definitions for seizure cycles will be necessary.

Cycles of seizures have been identified both from self-reported and electrographic events. Markers of cyclic activity have been captured from EEG signals and other non-invasive physiological measurements. How these physiological cycles are fundamentally linked to the cycles of seizure activity (such as in Figure 2) is still yet to be uncovered. The interaction of cycles and epilepsy therapies, such as ASMs, also remains to be investigated.

Beyond the scientific boundaries of our understanding of seizure cycles, there still exists a communication gap between the clinical and data science worlds with respect to the cyclic nature of seizure timing. We hope these academic communities continue to strive to find a common language and cross-disciplinary definitions to advance the field of seizure cycles in close collaboration.

## Author Contributions

PK, DF, BB, and EN developed the original concept and design of the manuscript, performed the literature search and analysis of data in the literature, contributed case studies, drafted, reviewed, and edited the manuscript. DE, RS, LL, and MM performed the literature search and analysis of data in the literature, contributed case studies, drafted, reviewed, and edited the manuscript. WD'S, MR, and MC participated in the interpretation of data in the literature, reviewed, and edited the manuscript for important intellectual content. All authors contributed to the article and approved the submitted version.

## Conflict of Interest

PK, DE, RS, EN, MM, DF, and MC have employment or financial interest in Seer Medical Pty. Ltd., which provides diagnostic EEG services. WD'S has employment or financial interest in KeyLead Health. WD'S and MC have employment or financial interest in Epi-Minder Pty. Ltd. MR has a research collaboration with UNEEG medical and has been a member of their advisory board. BB has a financial interest in Cadence Neurosciences Inc., and has received nonfinancial research support (devices for a study) from Medtronic Inc. The remaining authors declare that the research was conducted in the absence of any commercial or financial relationships that could be construed as a potential conflict of interest.

## Publisher's Note

All claims expressed in this article are solely those of the authors and do not necessarily represent those of their affiliated organizations, or those of the publisher, the editors and the reviewers. Any product that may be evaluated in this article, or claim that may be made by its manufacturer, is not guaranteed or endorsed by the publisher.
